# Tris(thio­cyanato-κ*N*)tris­(triphenyl­phosphine oxide-κ*O*)terbium(III)

**DOI:** 10.1107/S1600536812047289

**Published:** 2012-11-24

**Authors:** Lam N. Pham, Anthony T. Thames, Frankie D. White, Kang Rui Xiang, Richard E. Sykora

**Affiliations:** a Department of Chemistry, University of South Alabama, Mobile, AL 36688, USA

## Abstract

The title compound, [Tb(NCS)_3_(C_18_H_15_OP)_3_], contains a six-coordinate Tb^II^ cation surrounded by three *O*-bound triphenyl­phosphine oxide ligands and three *N*-bound thio­cyanate ligands, each in a *fac* arrangement. There are two crystallographically unique Tb^III^ atoms in the asymmetric unit. One Tb^III^ atom resides on a threefold rotation axis, while the other has no imposed crystallographic symmetry. The thio­cyanate ligands are bound through N atoms, illustrating the hard–hard bonding principles of metal complex chemistry.

## Related literature

For information on structures of related lanthanide phosphine oxide complexes, see: Bowden *et al.* (2012 ▶); Feazell *et al.* (2004 ▶). For the synthesis and characterization of lanthanide triphenyl­phosphine oxides with nitrate and thio­cyanate anions, see: Cousins & Hart (1967 ▶, 1968 ▶). For more information on the sizes of lanthanide ions, see: Brown & Altermatt (1985 ▶).
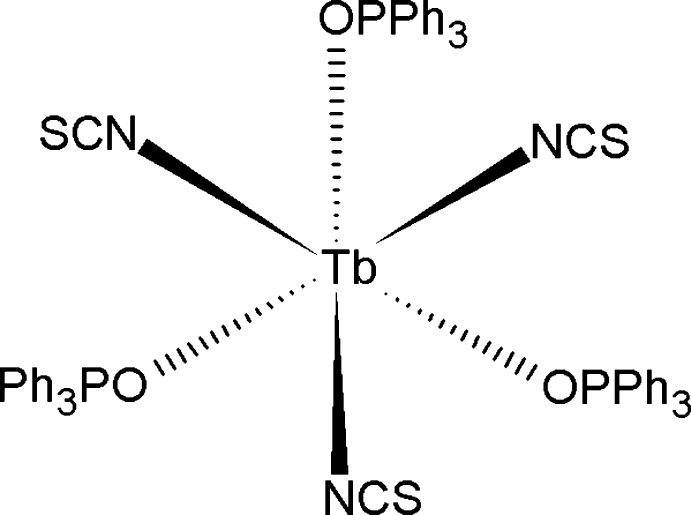


## Experimental

### 

#### Crystal data

[Tb(NCS)_3_(C_18_H_15_OP)_3_]
*M* *_r_* = 1168.01
Trigonal, 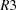

*a* = 38.6774 (5) Å
*c* = 12.3956 (2) Å
*V* = 16058.8 (4) Å^3^
*Z* = 12
Mo *K*α radiation
μ = 1.57 mm^−1^
*T* = 180 K
0.17 × 0.12 × 0.05 mm

#### Data collection

Agilent Xcalibur Eos CCD diffractometer
Absorption correction: multi-scan (*CrysAlis PRO*; Agilent, 2012 ▶) *T*_min_ = 0.913, *T*_max_ = 1.000
79947 measured reflections
13045 independent reflections
11605 reflections with *I* > 2σ(*I*)
*R*_int_ = 0.034

#### Refinement

*R*[*F* ^2^ > 2σ(*F* ^2^)] = 0.026
*wR*(*F* ^2^) = 0.056
*S* = 1.06
13045 reflections
841 parameters
1 restraint
H-atom parameters constrained
Δρ_max_ = 0.46 e Å^−3^
Δρ_min_ = −0.51 e Å^−3^
Absolute structure: Flack (1983 ▶), 6522 Friedel pairs
Flack parameter: −0.028 (4)

### 

Data collection: *CrysAlis PRO* (Agilent, 2012 ▶); cell refinement: *CrysAlis PRO*; data reduction: *CrysAlis PRO*; program(s) used to solve structure: *SHELXS97* (Sheldrick, 2008 ▶); program(s) used to refine structure: *SHELXL97* (Sheldrick, 2008 ▶); molecular graphics: *OLEX2* (Dolomanov *et al.*, 2009 ▶); software used to prepare material for publication: *publCIF* (Westrip, 2010 ▶).

## Supplementary Material

Crystal structure: contains datablock(s) global. DOI: 10.1107/S1600536812047289/hy2605sup1.cif

Additional supplementary materials:  crystallographic information; 3D view; checkCIF report

## Figures and Tables

**Table 1:** Selected bond lengths (Å)

Tb1—O1	2.265 (3)
Tb1—O2	2.246 (3)
Tb1—O3	2.243 (3)
Tb1—N1	2.350 (4)
Tb1—N2	2.359 (4)
Tb1—N3	2.356 (4)
Tb2—N4	2.355 (4)
Tb2—O4	2.267 (3)

**Fig. 1.**
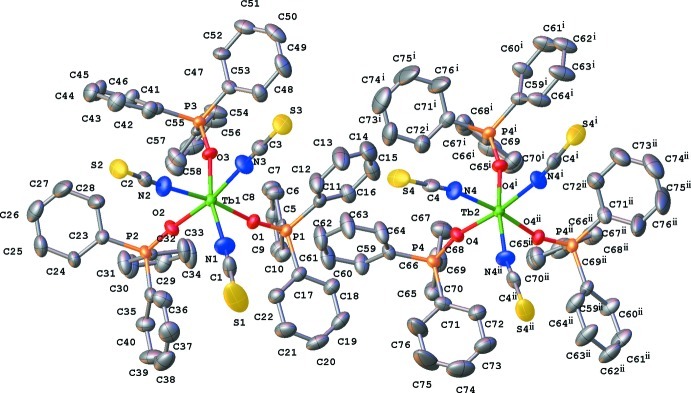
The molecular structure of the title compound. Displacement ellipsoids are drawn at the 50% probability level. H atoms have been omitted for clarity. [Symmetry codes: (i) 1-y, 1+x-y, z; (ii) -x+y, 1-x, z.]

## supplementary crystallographic information

### Comment

Lanthanide complexes containing triphenylphosphine oxides have been studied previously (Bowden *et al.*, 2012; Cousins & Hart, 1967, 1968; Feazell *et al.*, 2004) and are particularly interesting due to their tendency to simultaneously present a large number of these bulky ligands. In complexes of the larger lanthanides, such as [Nd(NCS)_3_{OP(C_6_H_5_)_3_}_4_], four triphenylphosphine oxide ligands are able to coordinate the metal center (Feazell *et al.*, 2004). With smallar lanthanide ions, such as Tb(III) in the title compound, [Tb(NCS)_3_{OP(C_6_H_5_)_3_}_3_], only three phosphine ligands are observed to coordinate. Even with a 1:4 mol ratio of terbium(III) nitrate to triphenylphosphine oxide in the synthetic strategy, there are only three triphenylphosphine oxide ligands that bind to the terbium center in the resultant products. In related lanthanide complexes containing triethylphosphine oxide ligands, the larger, lighter lanthanide ions contain three phosphine ligands and the smaller, heavier lanthanide ions contain only two ligands (Bowden *et al.*, 2012), which is also consistent with the trend observed in structural reports in the triphenylphosphine oxide complexes reported earlier and reported here. Interestingly, it seems apparent that the anhydrous nature of the lanthanide triphenylphosphine oxide systems are dominant as the title compound and past structural reports alike show this behaviour.

### Experimental

An ethanol solution of terbium(III) nitrate hydrate (1 mmol) and potassium thiocyanate (5 mmol) were combined. The precipitated KNO_3_ was removed by filtration from the reaction mixture and was discarded. After filtration, the resulting mixture was combined with an ethanol solution of triphenylphosphine oxide (4 mmol). The solution volume was reduced and the title compound crystallized as colourless single Tris(thiocyanato-κ*N*)tris(triphenylphosphine oxide-κ*O*)terbium(III)crystals from the solution after approximately one hour. No recrystallization was needed.

### Refinement

H atoms were placed in calculated positions and allowed to ride during subsequent refinement, with C—H = 0.93 Å and *U*_iso_(H) = 1.2*U*_eq_(C).

### Crystal data

**Table:**

[Tb(NCS)_3_(C_18_H_15_OP)_3_]	*D*_x_ = 1.449 Mg m^−^^3^
*M**_r_* = 1168.01	Mo *K*α radiation, λ = 0.71073 Å
Trigonal, *R*3	Cell parameters from 21753 reflections
Hall symbol: R 3	θ = 3.1–25.3°
*a* = 38.6774 (5) Å	µ = 1.57 mm^−^^1^
*c* = 12.3956 (2) Å	*T* = 180 K
*V* = 16058.8 (4) Å^3^	Prism, colourless
*Z* = 12	0.17 × 0.12 × 0.05 mm
*F*(000) = 7080	

### Data collection

**Table:**

Agilent Xcalibur Eos CCD diffractometer	13045 independent reflections
Radiation source: Enhance (Mo) X-ray Source	11605 reflections with *I* > 2σ(*I*)
Graphite monochromator	*R*_int_ = 0.034
Detector resolution: 16.0514 pixels mm^-1^	θ_max_ = 25.4°, θ_min_ = 3.1°
ω scans	*h* = −46→46
Absorption correction: multi-scan (*CrysAlis PRO*; Agilent, 2012)	*k* = −46→46
*T*_min_ = 0.913, *T*_max_ = 1.000	*l* = −14→14
79947 measured reflections	

### Refinement

**Table:**

Refinement on *F*^2^	Secondary atom site location: difference Fourier map
Least-squares matrix: full	Hydrogen site location: inferred from neighbouring sites
*R*[*F*^2^ > 2σ(*F*^2^)] = 0.026	H-atom parameters constrained
*wR*(*F*^2^) = 0.056	*w* = 1/[σ^2^(*F*_o_^2^) + (0.0195*P*)^2^ + 17.3876*P*] where *P* = (*F*_o_^2^ + 2*F*_c_^2^)/3
*S* = 1.06	(Δ/σ)_max_ = 0.002
13045 reflections	Δρ_max_ = 0.46 e Å^−^^3^
841 parameters	Δρ_min_ = −0.51 e Å^−^^3^
1 restraint	Absolute structure: Flack (1983), 6522 Friedel pairs
Primary atom site location: structure-invariant direct methods	Flack parameter: −0.028 (4)

### Special details

**Table:**

Geometry. All e.s.d.'s (except the e.s.d. in the dihedral angle between two l.s. planes) are estimated using the full covariance matrix. The cell e.s.d.'s are taken into account individually in the estimation of e.s.d.'s in distances, angles and torsion angles; correlations between e.s.d.'s in cell parameters are only used when they are defined by crystal symmetry. An approximate (isotropic) treatment of cell e.s.d.'s is used for estimating e.s.d.'s involving l.s. planes.

### Fractional atomic coordinates and isotropic or equivalent isotropic displacement parameters (Å^2^)

**Table:**

	*x*	*y*	*z*	*U*_iso_*/*U*_eq_	
Tb1	0.497674 (4)	0.499999 (4)	0.446699 (13)	0.02628 (5)	
Tb2	0.3333	0.6667	0.13775 (2)	0.02520 (11)	
P3	0.58595 (3)	0.57069 (3)	0.60883 (7)	0.0274 (2)	
P1	0.42911 (3)	0.52091 (3)	0.61339 (8)	0.0291 (2)	
P4	0.31745 (3)	0.57934 (3)	0.30108 (8)	0.0298 (2)	
S3	0.53514 (4)	0.61380 (4)	0.17730 (10)	0.0639 (3)	
S2	0.57413 (4)	0.46737 (4)	0.16746 (10)	0.0593 (3)	
S4	0.41608 (4)	0.63107 (4)	−0.11001 (11)	0.0747 (4)	
C4	0.39288 (12)	0.64579 (12)	−0.0310 (3)	0.0373 (11)	
C51	0.65967 (13)	0.69068 (12)	0.5925 (3)	0.0546 (11)	
H51	0.6854	0.7092	0.6147	0.066*	
O1	0.45464 (8)	0.50789 (8)	0.5536 (2)	0.0358 (7)	
O3	0.54690 (7)	0.54550 (8)	0.5507 (2)	0.0361 (7)	
N4	0.37642 (12)	0.65641 (12)	0.0250 (3)	0.0459 (10)	
O4	0.33202 (8)	0.61932 (7)	0.2483 (2)	0.0363 (7)	
P2	0.47985 (3)	0.41103 (3)	0.60768 (8)	0.0300 (2)	
O2	0.49021 (9)	0.44989 (8)	0.5539 (2)	0.0402 (8)	
C65	0.31722 (10)	0.58382 (10)	0.4444 (3)	0.0333 (8)	
S1	0.38132 (6)	0.41797 (5)	0.19602 (16)	0.1286 (8)	
C71	0.26838 (11)	0.54414 (10)	0.2577 (3)	0.0389 (9)	
C22	0.36604 (11)	0.45058 (11)	0.5413 (3)	0.0436 (9)	
H22	0.3854	0.4445	0.5200	0.052*	
C17	0.37722 (10)	0.48661 (11)	0.5903 (3)	0.0347 (8)	
C18	0.34790 (10)	0.49577 (12)	0.6215 (3)	0.0433 (9)	
H18	0.3553	0.5204	0.6527	0.052*	
C41	0.62123 (9)	0.55611 (9)	0.5662 (3)	0.0288 (7)	
C47	0.60574 (10)	0.62262 (10)	0.5790 (3)	0.0332 (8)	
C5	0.43799 (10)	0.52439 (10)	0.7551 (3)	0.0320 (8)	
C11	0.44195 (10)	0.57042 (10)	0.5705 (3)	0.0347 (8)	
C53	0.57978 (9)	0.56384 (10)	0.7520 (3)	0.0319 (8)	
C29	0.47584 (10)	0.41461 (10)	0.7501 (3)	0.0311 (8)	
C36	0.42050 (13)	0.37379 (12)	0.4573 (3)	0.0546 (11)	
H36	0.4380	0.3937	0.4105	0.065*	
C35	0.43226 (10)	0.37226 (10)	0.5611 (3)	0.0349 (8)	
C23	0.51747 (10)	0.39792 (10)	0.5858 (3)	0.0340 (8)	
C12	0.46025 (13)	0.58369 (13)	0.4722 (4)	0.0579 (12)	
H12	0.4643	0.5666	0.4280	0.069*	
C42	0.62219 (11)	0.54830 (12)	0.4578 (3)	0.0432 (9)	
H42	0.6061	0.5522	0.4093	0.052*	
C43	0.64722 (12)	0.53453 (13)	0.4213 (3)	0.0517 (11)	
H43	0.6479	0.5293	0.3484	0.062*	
C48	0.58323 (12)	0.63523 (11)	0.5240 (3)	0.0456 (10)	
H48	0.5577	0.6168	0.5004	0.055*	
N3	0.51559 (12)	0.55353 (11)	0.3266 (3)	0.0503 (10)	
C59	0.34993 (11)	0.55985 (10)	0.2705 (3)	0.0347 (8)	
C44	0.67081 (12)	0.52877 (13)	0.4930 (4)	0.0538 (11)	
H44	0.6872	0.5191	0.4689	0.065*	
C58	0.56276 (13)	0.52526 (11)	0.7911 (3)	0.0547 (11)	
H58	0.5549	0.5041	0.7434	0.066*	
C66	0.34695 (12)	0.61711 (11)	0.4941 (3)	0.0504 (10)	
H66	0.3661	0.6380	0.4528	0.060*	
C70	0.28860 (11)	0.55285 (11)	0.5085 (3)	0.0398 (9)	
H70	0.2682	0.5301	0.4762	0.048*	
C46	0.64555 (11)	0.55085 (11)	0.6375 (3)	0.0427 (9)	
H46	0.6453	0.5565	0.7104	0.051*	
C20	0.29724 (12)	0.43215 (14)	0.5569 (3)	0.0583 (12)	
H20	0.2704	0.4136	0.5461	0.070*	
C16	0.43541 (12)	0.59601 (11)	0.6347 (3)	0.0482 (10)	
H16	0.4227	0.5872	0.7009	0.058*	
C50	0.63667 (15)	0.70303 (12)	0.5393 (3)	0.0610 (12)	
H50	0.6467	0.7301	0.5270	0.073*	
C6	0.47519 (11)	0.55264 (11)	0.7945 (3)	0.0450 (9)	
H6	0.4951	0.5693	0.7464	0.054*	
C19	0.30802 (11)	0.46814 (14)	0.6059 (3)	0.0527 (11)	
H19	0.2885	0.4738	0.6285	0.063*	
C52	0.64440 (11)	0.65055 (10)	0.6127 (3)	0.0449 (9)	
H52	0.6598	0.6420	0.6489	0.054*	
C67	0.34860 (14)	0.61969 (14)	0.6052 (4)	0.0649 (13)	
H67	0.3691	0.6423	0.6379	0.078*	
C21	0.32615 (12)	0.42350 (13)	0.5238 (4)	0.0609 (12)	
H21	0.3188	0.3994	0.4895	0.073*	
C37	0.38306 (15)	0.34607 (15)	0.4226 (4)	0.0675 (14)	
H37	0.3754	0.3474	0.3523	0.081*	
C9	0.41732 (12)	0.50365 (12)	0.9381 (3)	0.0462 (10)	
H9	0.3979	0.4871	0.9872	0.055*	
C3	0.52387 (12)	0.57841 (12)	0.2642 (3)	0.0400 (10)	
C72	0.24116 (11)	0.55670 (12)	0.2374 (3)	0.0540 (11)	
H72	0.2487	0.5834	0.2470	0.065*	
C8	0.45469 (12)	0.53253 (12)	0.9747 (3)	0.0453 (10)	
H8	0.4603	0.5355	1.0482	0.054*	
C61	0.37610 (14)	0.51666 (13)	0.3076 (3)	0.0597 (12)	
H61	0.3769	0.4975	0.3511	0.072*	
C45	0.67034 (12)	0.53714 (13)	0.6000 (3)	0.0540 (11)	
H45	0.6868	0.5336	0.6481	0.065*	
C40	0.40580 (11)	0.34186 (11)	0.6291 (3)	0.0492 (10)	
H40	0.4134	0.3404	0.6994	0.059*	
N1	0.44182 (12)	0.46278 (12)	0.3357 (3)	0.0567 (11)	
C10	0.40893 (11)	0.49949 (11)	0.8300 (3)	0.0411 (9)	
H10	0.3838	0.4801	0.8061	0.049*	
C38	0.35679 (14)	0.31654 (16)	0.4891 (4)	0.0701 (14)	
H38	0.3313	0.2983	0.4650	0.084*	
C60	0.35217 (12)	0.53192 (12)	0.3363 (3)	0.0501 (10)	
H60	0.3374	0.5236	0.3998	0.060*	
C39	0.36843 (13)	0.31391 (14)	0.5927 (4)	0.0704 (14)	
H39	0.3511	0.2933	0.6379	0.085*	
C24	0.50903 (11)	0.35862 (11)	0.5887 (3)	0.0421 (9)	
H24	0.4827	0.3379	0.5915	0.050*	
C14	0.46665 (15)	0.64753 (14)	0.5032 (5)	0.0735 (15)	
H14	0.4754	0.6736	0.4812	0.088*	
C68	0.32109 (15)	0.59001 (14)	0.6672 (3)	0.0607 (12)	
H68	0.3226	0.5924	0.7419	0.073*	
C32	0.46498 (13)	0.41878 (14)	0.9697 (3)	0.0567 (11)	
H32	0.4614	0.4205	1.0433	0.068*	
C30	0.48218 (13)	0.39124 (13)	0.8237 (3)	0.0570 (12)	
H30	0.4904	0.3739	0.7987	0.068*	
C25	0.53996 (14)	0.35029 (13)	0.5874 (3)	0.0562 (11)	
H25	0.5342	0.3239	0.5902	0.067*	
C69	0.29066 (13)	0.55606 (13)	0.6193 (3)	0.0532 (11)	
H69	0.2717	0.5355	0.6617	0.064*	
C31	0.47658 (13)	0.39319 (14)	0.9316 (3)	0.0608 (12)	
H31	0.4807	0.3770	0.9792	0.073*	
C34	0.46363 (14)	0.43976 (13)	0.7911 (3)	0.0587 (12)	
H34	0.4587	0.4556	0.7445	0.070*	
C7	0.48309 (12)	0.55644 (12)	0.9024 (3)	0.0515 (10)	
H7	0.5082	0.5756	0.9269	0.062*	
C26	0.57884 (13)	0.38046 (15)	0.5822 (3)	0.0613 (12)	
H26	0.5994	0.3746	0.5818	0.074*	
C15	0.44761 (15)	0.63415 (13)	0.6011 (4)	0.0639 (13)	
H15	0.4430	0.6511	0.6445	0.077*	
C63	0.39710 (16)	0.55732 (14)	0.1507 (4)	0.0704 (15)	
H63	0.4125	0.5661	0.0884	0.084*	
C1	0.41704 (15)	0.44470 (13)	0.2774 (4)	0.0501 (13)	
C62	0.39892 (15)	0.52932 (13)	0.2156 (3)	0.0629 (13)	
H62	0.4154	0.5191	0.1974	0.075*	
C2	0.55123 (12)	0.47549 (11)	0.2650 (3)	0.0417 (10)	
N2	0.53486 (12)	0.48160 (12)	0.3335 (3)	0.0502 (10)	
C28	0.55682 (12)	0.42797 (12)	0.5777 (4)	0.0594 (12)	
H28	0.5629	0.4544	0.5724	0.071*	
C49	0.59883 (16)	0.67590 (13)	0.5037 (3)	0.0626 (13)	
H49	0.5838	0.6846	0.4664	0.075*	
C64	0.37262 (14)	0.57240 (13)	0.1772 (3)	0.0537 (12)	
H64	0.3713	0.5910	0.1325	0.064*	
C54	0.59052 (12)	0.59435 (11)	0.8253 (3)	0.0553 (11)	
H54	0.6018	0.6205	0.8009	0.066*	
C55	0.58479 (13)	0.58651 (13)	0.9335 (3)	0.0692 (14)	
H55	0.5919	0.6074	0.9819	0.083*	
C73	0.20311 (13)	0.52994 (15)	0.2033 (4)	0.0685 (13)	
H73	0.1852	0.5388	0.1895	0.082*	
C56	0.56874 (12)	0.54826 (14)	0.9710 (3)	0.0600 (12)	
H56	0.5657	0.5431	1.0447	0.072*	
C57	0.55741 (14)	0.51801 (14)	0.8996 (4)	0.0641 (13)	
H57	0.5458	0.4919	0.9246	0.077*	
C74	0.19151 (16)	0.49143 (18)	0.1899 (4)	0.0869 (18)	
H74	0.1654	0.4734	0.1693	0.104*	
C27	0.58739 (13)	0.41903 (16)	0.5775 (4)	0.0768 (16)	
H27	0.6138	0.4395	0.5741	0.092*	
C33	0.45873 (17)	0.44177 (15)	0.8990 (4)	0.0787 (16)	
H33	0.4509	0.4594	0.9249	0.094*	
C76	0.25688 (15)	0.50466 (13)	0.2384 (5)	0.0909 (19)	
H76	0.2752	0.4959	0.2469	0.109*	
C13	0.47266 (16)	0.62224 (15)	0.4385 (5)	0.0814 (17)	
H13	0.4851	0.6310	0.3718	0.098*	
C75	0.21866 (18)	0.47841 (15)	0.2070 (6)	0.126 (3)	
H75	0.2108	0.4516	0.1969	0.151*	

### Atomic displacement parameters (Å^2^)

**Table:**

	*U*^11^	*U*^22^	*U*^33^	*U*^12^	*U*^13^	*U*^23^
Tb1	0.02646 (11)	0.02665 (13)	0.02659 (10)	0.01392 (11)	0.00029 (10)	0.00162 (9)
Tb2	0.02401 (13)	0.02401 (13)	0.0276 (2)	0.01201 (6)	0.000	0.000
P3	0.0224 (5)	0.0253 (5)	0.0320 (5)	0.0101 (4)	−0.0032 (4)	0.0014 (4)
P1	0.0245 (5)	0.0304 (5)	0.0344 (5)	0.0153 (4)	0.0018 (4)	−0.0009 (4)
P4	0.0316 (5)	0.0243 (5)	0.0315 (5)	0.0124 (4)	−0.0007 (4)	0.0037 (4)
S3	0.0716 (8)	0.0649 (8)	0.0577 (7)	0.0359 (7)	0.0134 (6)	0.0363 (6)
S2	0.0656 (8)	0.0551 (7)	0.0596 (8)	0.0320 (6)	0.0266 (6)	−0.0029 (6)
S4	0.0617 (8)	0.0616 (8)	0.0860 (9)	0.0198 (6)	0.0362 (7)	−0.0226 (7)
C4	0.034 (2)	0.035 (2)	0.031 (2)	0.0081 (18)	0.0123 (18)	0.0039 (17)
C51	0.055 (3)	0.036 (2)	0.052 (3)	0.007 (2)	0.012 (2)	0.0042 (19)
O1	0.0308 (14)	0.0394 (16)	0.0413 (18)	0.0206 (13)	−0.0008 (12)	−0.0040 (13)
O3	0.0229 (13)	0.0362 (15)	0.0427 (17)	0.0099 (12)	−0.0065 (12)	0.0011 (12)
N4	0.048 (2)	0.061 (3)	0.038 (2)	0.034 (2)	0.0204 (18)	0.0076 (19)
O4	0.0411 (16)	0.0284 (14)	0.0406 (16)	0.0183 (12)	0.0045 (12)	0.0061 (12)
P2	0.0327 (5)	0.0246 (5)	0.0356 (6)	0.0165 (4)	0.0054 (4)	0.0063 (4)
O2	0.0499 (18)	0.0292 (15)	0.0436 (19)	0.0214 (13)	0.0081 (14)	0.0088 (13)
C65	0.0273 (18)	0.0238 (18)	0.050 (2)	0.0139 (16)	0.0000 (16)	0.0050 (16)
S1	0.1507 (17)	0.0882 (12)	0.1559 (17)	0.0665 (12)	−0.1274 (15)	−0.0623 (11)
C71	0.040 (2)	0.035 (2)	0.0317 (19)	0.0114 (17)	−0.0074 (16)	0.0038 (15)
C22	0.037 (2)	0.044 (2)	0.048 (2)	0.0186 (19)	0.0021 (18)	−0.0053 (18)
C17	0.0295 (19)	0.046 (2)	0.0294 (18)	0.0193 (17)	0.0009 (15)	0.0046 (16)
C18	0.038 (2)	0.050 (2)	0.043 (2)	0.0233 (19)	−0.0007 (17)	−0.0016 (18)
C41	0.0263 (17)	0.0248 (17)	0.0325 (18)	0.0106 (14)	−0.0010 (14)	−0.0012 (14)
C47	0.036 (2)	0.0309 (19)	0.0297 (18)	0.0148 (16)	0.0064 (15)	0.0048 (14)
C5	0.0260 (18)	0.0338 (19)	0.045 (2)	0.0218 (16)	−0.0007 (16)	−0.0080 (16)
C11	0.0281 (18)	0.036 (2)	0.042 (2)	0.0180 (16)	0.0007 (15)	0.0035 (16)
C53	0.0180 (17)	0.0313 (19)	0.043 (2)	0.0099 (15)	0.0009 (15)	0.0067 (16)
C29	0.0235 (18)	0.0271 (19)	0.043 (2)	0.0126 (16)	0.0039 (15)	0.0055 (16)
C36	0.062 (3)	0.048 (3)	0.047 (3)	0.022 (2)	−0.007 (2)	0.002 (2)
C35	0.038 (2)	0.0324 (19)	0.041 (2)	0.0218 (17)	0.0045 (16)	0.0015 (16)
C23	0.0342 (19)	0.036 (2)	0.0346 (19)	0.0199 (17)	0.0069 (15)	0.0036 (15)
C12	0.069 (3)	0.059 (3)	0.063 (3)	0.045 (3)	0.028 (2)	0.022 (2)
C42	0.043 (2)	0.055 (3)	0.036 (2)	0.028 (2)	−0.0060 (17)	−0.0014 (18)
C43	0.053 (3)	0.067 (3)	0.040 (2)	0.034 (2)	−0.002 (2)	−0.009 (2)
C48	0.057 (3)	0.042 (2)	0.044 (2)	0.028 (2)	−0.010 (2)	−0.0026 (18)
N3	0.070 (3)	0.047 (2)	0.034 (2)	0.029 (2)	0.0062 (19)	0.0161 (17)
C59	0.046 (2)	0.0294 (19)	0.0328 (19)	0.0219 (17)	0.0011 (16)	0.0016 (15)
C44	0.050 (3)	0.060 (3)	0.060 (3)	0.034 (2)	0.007 (2)	−0.003 (2)
C58	0.085 (3)	0.036 (2)	0.042 (2)	0.029 (2)	0.008 (2)	0.0042 (18)
C66	0.051 (2)	0.041 (2)	0.042 (2)	0.011 (2)	−0.0079 (19)	−0.0028 (18)
C70	0.039 (2)	0.044 (2)	0.036 (2)	0.0202 (18)	0.0063 (16)	0.0088 (16)
C46	0.043 (2)	0.059 (3)	0.034 (2)	0.032 (2)	−0.0090 (17)	−0.0064 (18)
C20	0.029 (2)	0.075 (3)	0.052 (3)	0.012 (2)	−0.0016 (19)	−0.001 (2)
C16	0.061 (3)	0.045 (2)	0.044 (2)	0.031 (2)	−0.0032 (19)	−0.0009 (18)
C50	0.093 (4)	0.032 (2)	0.048 (3)	0.024 (2)	0.011 (2)	0.0078 (19)
C6	0.036 (2)	0.043 (2)	0.046 (2)	0.0121 (18)	−0.0032 (17)	0.0041 (18)
C19	0.034 (2)	0.078 (3)	0.049 (2)	0.031 (2)	0.0008 (18)	0.003 (2)
C52	0.033 (2)	0.031 (2)	0.057 (2)	0.0065 (17)	0.0045 (18)	0.0075 (18)
C67	0.072 (3)	0.052 (3)	0.057 (3)	0.020 (3)	−0.019 (3)	−0.008 (2)
C21	0.044 (3)	0.055 (3)	0.066 (3)	0.011 (2)	−0.005 (2)	−0.015 (2)
C37	0.071 (4)	0.073 (4)	0.058 (3)	0.036 (3)	−0.024 (3)	−0.011 (3)
C9	0.047 (2)	0.054 (3)	0.038 (2)	0.026 (2)	0.0035 (18)	−0.0012 (18)
C3	0.038 (2)	0.044 (2)	0.037 (2)	0.020 (2)	0.0037 (17)	0.0036 (19)
C72	0.042 (2)	0.049 (3)	0.067 (3)	0.020 (2)	0.003 (2)	0.005 (2)
C8	0.054 (3)	0.056 (3)	0.037 (2)	0.036 (2)	−0.0105 (19)	−0.0101 (19)
C61	0.095 (4)	0.067 (3)	0.045 (2)	0.061 (3)	0.004 (2)	0.016 (2)
C45	0.048 (2)	0.076 (3)	0.054 (3)	0.043 (2)	−0.009 (2)	−0.005 (2)
C40	0.044 (2)	0.046 (2)	0.048 (2)	0.015 (2)	0.0056 (19)	0.0000 (19)
N1	0.052 (2)	0.059 (3)	0.049 (2)	0.020 (2)	−0.021 (2)	−0.009 (2)
C10	0.035 (2)	0.048 (2)	0.039 (2)	0.0201 (19)	0.0016 (16)	−0.0058 (17)
C38	0.046 (3)	0.088 (4)	0.075 (4)	0.032 (3)	−0.017 (2)	−0.032 (3)
C60	0.072 (3)	0.061 (3)	0.037 (2)	0.047 (2)	0.0126 (19)	0.0192 (19)
C39	0.045 (3)	0.060 (3)	0.075 (3)	0.002 (2)	0.016 (2)	−0.007 (3)
C24	0.043 (2)	0.034 (2)	0.056 (2)	0.0246 (18)	−0.0016 (18)	−0.0013 (17)
C14	0.076 (4)	0.050 (3)	0.101 (4)	0.036 (3)	−0.001 (3)	0.021 (3)
C68	0.095 (4)	0.077 (3)	0.031 (2)	0.058 (3)	−0.008 (2)	−0.003 (2)
C32	0.062 (3)	0.082 (3)	0.040 (2)	0.047 (3)	0.004 (2)	−0.004 (2)
C30	0.092 (3)	0.070 (3)	0.039 (2)	0.063 (3)	0.013 (2)	0.014 (2)
C25	0.073 (3)	0.056 (3)	0.062 (3)	0.049 (3)	−0.003 (2)	−0.006 (2)
C69	0.058 (3)	0.062 (3)	0.044 (2)	0.034 (2)	0.012 (2)	0.015 (2)
C31	0.081 (3)	0.084 (3)	0.043 (2)	0.061 (3)	0.015 (2)	0.020 (2)
C34	0.094 (4)	0.067 (3)	0.049 (3)	0.065 (3)	−0.005 (2)	−0.004 (2)
C7	0.041 (2)	0.050 (3)	0.055 (3)	0.016 (2)	−0.017 (2)	−0.006 (2)
C26	0.054 (3)	0.089 (4)	0.060 (3)	0.050 (3)	−0.001 (2)	−0.016 (3)
C15	0.090 (4)	0.053 (3)	0.065 (3)	0.048 (3)	−0.020 (3)	−0.012 (2)
C63	0.117 (4)	0.076 (3)	0.055 (3)	0.076 (3)	0.040 (3)	0.026 (2)
C1	0.068 (3)	0.055 (3)	0.047 (3)	0.045 (3)	−0.023 (2)	−0.018 (2)
C62	0.099 (4)	0.072 (3)	0.053 (3)	0.069 (3)	0.015 (2)	0.009 (2)
C2	0.046 (3)	0.032 (2)	0.049 (3)	0.020 (2)	0.000 (2)	−0.0019 (18)
N2	0.058 (2)	0.065 (3)	0.041 (2)	0.041 (2)	0.0095 (18)	−0.0047 (19)
C28	0.038 (2)	0.040 (2)	0.094 (3)	0.015 (2)	0.019 (2)	0.000 (2)
C49	0.101 (4)	0.052 (3)	0.052 (3)	0.051 (3)	−0.006 (3)	0.010 (2)
C64	0.089 (3)	0.053 (3)	0.040 (2)	0.052 (3)	0.019 (2)	0.014 (2)
C54	0.059 (3)	0.033 (2)	0.044 (2)	0.001 (2)	0.002 (2)	0.0010 (18)
C55	0.069 (3)	0.055 (3)	0.041 (2)	−0.001 (2)	0.005 (2)	−0.007 (2)
C73	0.039 (3)	0.074 (3)	0.077 (3)	0.017 (2)	−0.009 (2)	0.007 (3)
C56	0.046 (3)	0.077 (3)	0.035 (2)	0.014 (2)	−0.0027 (19)	0.008 (2)
C57	0.089 (4)	0.054 (3)	0.054 (3)	0.040 (3)	0.015 (3)	0.020 (2)
C74	0.057 (3)	0.080 (4)	0.081 (4)	0.003 (3)	−0.029 (3)	−0.004 (3)
C27	0.037 (2)	0.071 (3)	0.117 (5)	0.023 (2)	0.014 (3)	−0.014 (3)
C33	0.135 (5)	0.094 (4)	0.057 (3)	0.095 (4)	0.003 (3)	−0.009 (3)
C76	0.081 (4)	0.039 (3)	0.143 (5)	0.023 (3)	−0.056 (4)	−0.024 (3)
C13	0.094 (4)	0.074 (4)	0.097 (4)	0.057 (3)	0.048 (3)	0.047 (3)
C75	0.104 (5)	0.041 (3)	0.205 (8)	0.015 (3)	−0.093 (5)	−0.033 (4)

### Geometric parameters (Å, º)

**Table:**

Tb1—O1	2.265 (3)	C20—C19	1.379 (6)
Tb1—O2	2.246 (3)	C20—C21	1.380 (6)
Tb1—O3	2.243 (3)	C16—H16	0.9300
Tb1—N1	2.350 (4)	C16—C15	1.369 (5)
Tb1—N2	2.359 (4)	C50—H50	0.9300
Tb1—N3	2.356 (4)	C50—C49	1.379 (6)
Tb2—N4	2.355 (4)	C6—H6	0.9300
Tb2—O4	2.267 (3)	C6—C7	1.364 (5)
P3—O3	1.509 (3)	C19—H19	0.9300
P3—C41	1.797 (3)	C52—H52	0.9300
P3—C47	1.794 (3)	C67—H67	0.9300
P3—C53	1.793 (4)	C67—C68	1.349 (6)
P1—O1	1.508 (3)	C21—H21	0.9300
P1—C17	1.791 (3)	C37—H37	0.9300
P1—C5	1.782 (4)	C37—C38	1.362 (7)
P1—C11	1.801 (4)	C9—H9	0.9300
P4—O4	1.505 (3)	C9—C8	1.389 (5)
P4—C65	1.786 (4)	C9—C10	1.369 (5)
P4—C71	1.778 (4)	C72—H72	0.9300
P4—C59	1.799 (4)	C72—C73	1.376 (5)
S3—C3	1.621 (4)	C8—H8	0.9300
S2—C2	1.619 (4)	C8—C7	1.360 (5)
S4—C4	1.613 (4)	C61—H61	0.9300
C4—N4	1.148 (5)	C61—C60	1.370 (5)
C51—H51	0.9300	C61—C62	1.374 (6)
C51—C50	1.371 (6)	C45—H45	0.9300
C51—C52	1.380 (5)	C40—H40	0.9300
P2—O2	1.504 (3)	C40—C39	1.378 (6)
P2—C29	1.784 (4)	N1—C1	1.123 (5)
P2—C35	1.792 (4)	C10—H10	0.9300
P2—C23	1.784 (3)	C38—H38	0.9300
C65—C66	1.371 (5)	C38—C39	1.381 (6)
C65—C70	1.402 (5)	C60—H60	0.9300
S1—C1	1.602 (5)	C39—H39	0.9300
C71—C72	1.385 (5)	C24—H24	0.9300
C71—C76	1.381 (5)	C24—C25	1.386 (5)
C22—H22	0.9300	C14—H14	0.9300
C22—C17	1.377 (5)	C14—C15	1.379 (7)
C22—C21	1.382 (5)	C14—C13	1.372 (7)
C17—C18	1.401 (5)	C68—H68	0.9300
C18—H18	0.9300	C68—C69	1.384 (6)
C18—C19	1.382 (5)	C32—H32	0.9300
C41—C42	1.381 (5)	C32—C31	1.360 (5)
C41—C46	1.378 (5)	C32—C33	1.353 (6)
C47—C48	1.373 (5)	C30—H30	0.9300
C47—C52	1.401 (5)	C30—C31	1.363 (5)
C5—C6	1.389 (5)	C25—H25	0.9300
C5—C10	1.404 (5)	C25—C26	1.368 (6)
C11—C12	1.374 (5)	C69—H69	0.9300
C11—C16	1.388 (5)	C31—H31	0.9300
C53—C58	1.383 (5)	C34—H34	0.9300
C53—C54	1.378 (5)	C34—C33	1.359 (6)
C29—C30	1.389 (5)	C7—H7	0.9300
C29—C34	1.374 (5)	C26—H26	0.9300
C36—H36	0.9300	C26—C27	1.358 (6)
C36—C35	1.376 (5)	C15—H15	0.9300
C36—C37	1.371 (6)	C63—H63	0.9300
C35—C40	1.391 (5)	C63—C62	1.379 (5)
C23—C24	1.386 (5)	C63—C64	1.377 (6)
C23—C28	1.382 (5)	C62—H62	0.9300
C12—H12	0.9300	C2—N2	1.152 (5)
C12—C13	1.383 (6)	C28—H28	0.9300
C42—H42	0.9300	C28—C27	1.388 (6)
C42—C43	1.393 (5)	C49—H49	0.9300
C43—H43	0.9300	C64—H64	0.9300
C43—C44	1.370 (6)	C54—H54	0.9300
C48—H48	0.9300	C54—C55	1.368 (5)
C48—C49	1.397 (5)	C55—H55	0.9300
N3—C3	1.148 (5)	C55—C56	1.368 (6)
C59—C60	1.390 (5)	C73—H73	0.9300
C59—C64	1.384 (5)	C73—C74	1.334 (7)
C44—H44	0.9300	C56—H56	0.9300
C44—C45	1.368 (6)	C56—C57	1.353 (6)
C58—H58	0.9300	C57—H57	0.9300
C58—C57	1.367 (5)	C74—H74	0.9300
C66—H66	0.9300	C74—C75	1.389 (8)
C66—C67	1.379 (6)	C27—H27	0.9300
C70—H70	0.9300	C33—H33	0.9300
C70—C69	1.377 (5)	C76—H76	0.9300
C46—H46	0.9300	C76—C75	1.366 (6)
C46—C45	1.387 (5)	C13—H13	0.9300
C20—H20	0.9300	C75—H75	0.9300
			
O1—Tb1—N3	99.48 (12)	C15—C16—H16	119.8
O1—Tb1—N1	84.99 (12)	C51—C50—H50	119.5
O1—Tb1—N2	171.36 (12)	C51—C50—C49	121.0 (4)
O3—Tb1—O1	87.18 (10)	C49—C50—H50	119.5
O3—Tb1—O2	91.69 (10)	C5—C6—H6	119.4
O3—Tb1—N3	84.97 (11)	C7—C6—C5	121.2 (4)
O3—Tb1—N1	167.59 (12)	C7—C6—H6	119.4
O3—Tb1—N2	99.90 (11)	C18—C19—H19	120.0
O2—Tb1—O1	89.08 (10)	C20—C19—C18	120.0 (4)
O2—Tb1—N3	170.63 (13)	C20—C19—H19	120.0
O2—Tb1—N1	97.79 (12)	C51—C52—C47	120.3 (4)
O2—Tb1—N2	85.77 (11)	C51—C52—H52	119.8
N3—Tb1—N2	86.18 (13)	C47—C52—H52	119.8
N1—Tb1—N3	86.84 (14)	C66—C67—H67	119.4
N1—Tb1—N2	88.83 (14)	C68—C67—C66	121.3 (4)
N4^i^—Tb2—N4^ii^	88.40 (14)	C68—C67—H67	119.4
N4^i^—Tb2—N4	88.41 (14)	C22—C21—H21	119.9
N4^ii^—Tb2—N4	88.41 (14)	C20—C21—C22	120.1 (4)
O4—Tb2—N4^ii^	97.42 (12)	C20—C21—H21	119.9
O4^i^—Tb2—N4^ii^	172.71 (11)	C36—C37—H37	119.3
O4^ii^—Tb2—N4	172.71 (11)	C38—C37—C36	121.3 (5)
O4^i^—Tb2—N4	97.42 (12)	C38—C37—H37	119.3
O4—Tb2—N4	87.38 (11)	C8—C9—H9	119.8
O4^ii^—Tb2—N4^i^	97.41 (12)	C10—C9—H9	119.8
O4^i^—Tb2—N4^i^	87.38 (11)	C10—C9—C8	120.3 (4)
O4—Tb2—N4^i^	172.71 (12)	N3—C3—S3	179.0 (4)
O4^ii^—Tb2—N4^ii^	87.38 (11)	C71—C72—H72	119.8
O4^ii^—Tb2—O4^i^	87.27 (10)	C73—C72—C71	120.4 (4)
O4^ii^—Tb2—O4	87.27 (10)	C73—C72—H72	119.8
O4^i^—Tb2—O4	87.27 (10)	C9—C8—H8	120.2
O3—P3—C41	109.19 (15)	C7—C8—C9	119.6 (4)
O3—P3—C47	110.67 (16)	C7—C8—H8	120.2
O3—P3—C53	111.18 (16)	C60—C61—H61	119.5
C47—P3—C41	108.95 (15)	C60—C61—C62	120.9 (4)
C53—P3—C41	108.10 (16)	C62—C61—H61	119.5
C53—P3—C47	108.68 (16)	C44—C45—C46	120.6 (4)
O1—P1—C17	110.64 (16)	C44—C45—H45	119.7
O1—P1—C5	112.06 (16)	C46—C45—H45	119.7
O1—P1—C11	109.36 (16)	C35—C40—H40	119.9
C17—P1—C11	110.58 (16)	C39—C40—C35	120.3 (4)
C5—P1—C17	108.41 (16)	C39—C40—H40	119.9
C5—P1—C11	105.69 (16)	C1—N1—Tb1	174.7 (4)
O4—P4—C65	110.52 (16)	C5—C10—H10	119.8
O4—P4—C71	111.29 (16)	C9—C10—C5	120.4 (4)
O4—P4—C59	110.95 (16)	C9—C10—H10	119.8
C65—P4—C59	106.78 (16)	C37—C38—H38	120.3
C71—P4—C65	108.68 (17)	C37—C38—C39	119.3 (4)
C71—P4—C59	108.47 (17)	C39—C38—H38	120.3
N4—C4—S4	179.7 (4)	C59—C60—H60	120.0
C50—C51—H51	120.3	C61—C60—C59	120.0 (4)
C50—C51—C52	119.5 (4)	C61—C60—H60	120.0
C52—C51—H51	120.3	C40—C39—C38	120.0 (4)
P1—O1—Tb1	168.67 (18)	C40—C39—H39	120.0
P3—O3—Tb1	164.49 (17)	C38—C39—H39	120.0
C4—N4—Tb2	170.0 (4)	C23—C24—H24	120.1
P4—O4—Tb2	159.75 (17)	C25—C24—C23	119.8 (4)
O2—P2—C29	110.55 (16)	C25—C24—H24	120.1
O2—P2—C35	110.03 (17)	C15—C14—H14	120.1
O2—P2—C23	111.66 (16)	C13—C14—H14	120.1
C29—P2—C35	106.91 (16)	C13—C14—C15	119.8 (4)
C29—P2—C23	106.70 (16)	C67—C68—H68	120.1
C23—P2—C35	110.84 (16)	C67—C68—C69	119.8 (4)
P2—O2—Tb1	168.4 (2)	C69—C68—H68	120.1
C66—C65—P4	119.7 (3)	C31—C32—H32	120.5
C66—C65—C70	118.8 (4)	C33—C32—H32	120.5
C70—C65—P4	121.4 (3)	C33—C32—C31	119.0 (4)
C72—C71—P4	119.6 (3)	C29—C30—H30	119.2
C76—C71—P4	122.0 (3)	C31—C30—C29	121.6 (4)
C76—C71—C72	118.4 (4)	C31—C30—H30	119.2
C17—C22—H22	119.9	C24—C25—H25	119.6
C17—C22—C21	120.2 (4)	C26—C25—C24	120.7 (4)
C21—C22—H22	119.9	C26—C25—H25	119.6
C22—C17—P1	119.3 (3)	C70—C69—C68	119.8 (4)
C22—C17—C18	119.6 (3)	C70—C69—H69	120.1
C18—C17—P1	121.2 (3)	C68—C69—H69	120.1
C17—C18—H18	120.1	C32—C31—C30	120.0 (4)
C19—C18—C17	119.8 (4)	C32—C31—H31	120.0
C19—C18—H18	120.1	C30—C31—H31	120.0
C42—C41—P3	117.5 (3)	C29—C34—H34	119.6
C46—C41—P3	122.6 (3)	C33—C34—C29	120.8 (4)
C46—C41—C42	119.8 (3)	C33—C34—H34	119.6
C48—C47—P3	120.6 (3)	C6—C7—H7	119.6
C48—C47—C52	119.7 (3)	C8—C7—C6	120.8 (4)
C52—C47—P3	119.7 (3)	C8—C7—H7	119.6
C6—C5—P1	119.4 (3)	C25—C26—H26	120.0
C6—C5—C10	117.8 (3)	C27—C26—C25	119.9 (4)
C10—C5—P1	122.9 (3)	C27—C26—H26	120.0
C12—C11—P1	118.4 (3)	C16—C15—C14	120.2 (4)
C12—C11—C16	119.1 (4)	C16—C15—H15	119.9
C16—C11—P1	122.5 (3)	C14—C15—H15	119.9
C58—C53—P3	117.6 (3)	C62—C63—H63	119.7
C54—C53—P3	124.4 (3)	C64—C63—H63	119.7
C54—C53—C58	118.0 (3)	C64—C63—C62	120.6 (4)
C30—C29—P2	123.3 (3)	N1—C1—S1	178.6 (5)
C34—C29—P2	119.7 (3)	C61—C62—C63	119.2 (4)
C34—C29—C30	116.9 (4)	C61—C62—H62	120.4
C35—C36—H36	119.9	C63—C62—H62	120.4
C37—C36—H36	119.9	N2—C2—S2	179.1 (4)
C37—C36—C35	120.1 (4)	C2—N2—Tb1	168.7 (3)
C36—C35—P2	119.2 (3)	C23—C28—H28	119.7
C36—C35—C40	118.9 (4)	C23—C28—C27	120.5 (4)
C40—C35—P2	121.7 (3)	C27—C28—H28	119.7
C24—C23—P2	121.9 (3)	C48—C49—H49	120.1
C28—C23—P2	118.9 (3)	C50—C49—C48	119.7 (4)
C28—C23—C24	118.7 (3)	C50—C49—H49	120.1
C11—C12—H12	119.8	C59—C64—H64	120.0
C11—C12—C13	120.5 (4)	C63—C64—C59	120.0 (4)
C13—C12—H12	119.8	C63—C64—H64	120.0
C41—C42—H42	120.0	C53—C54—H54	119.7
C41—C42—C43	120.0 (3)	C55—C54—C53	120.7 (4)
C43—C42—H42	120.0	C55—C54—H54	119.7
C42—C43—H43	120.1	C54—C55—H55	119.7
C44—C43—C42	119.8 (4)	C56—C55—C54	120.5 (4)
C44—C43—H43	120.1	C56—C55—H55	119.7
C47—C48—H48	120.1	C72—C73—H73	119.5
C47—C48—C49	119.7 (4)	C74—C73—C72	121.0 (5)
C49—C48—H48	120.1	C74—C73—H73	119.5
C3—N3—Tb1	176.9 (3)	C55—C56—H56	120.4
C60—C59—P4	122.0 (3)	C57—C56—C55	119.2 (4)
C64—C59—P4	118.8 (3)	C57—C56—H56	120.4
C64—C59—C60	119.2 (3)	C58—C57—H57	119.5
C43—C44—H44	119.9	C56—C57—C58	121.0 (4)
C45—C44—C43	120.2 (4)	C56—C57—H57	119.5
C45—C44—H44	119.9	C73—C74—H74	120.2
C53—C58—H58	119.8	C73—C74—C75	119.5 (5)
C57—C58—C53	120.5 (4)	C75—C74—H74	120.2
C57—C58—H58	119.8	C26—C27—C28	120.2 (4)
C65—C66—H66	119.9	C26—C27—H27	119.9
C65—C66—C67	120.2 (4)	C28—C27—H27	119.9
C67—C66—H66	119.9	C32—C33—C34	121.6 (4)
C65—C70—H70	119.9	C32—C33—H33	119.2
C69—C70—C65	120.2 (4)	C34—C33—H33	119.2
C69—C70—H70	119.9	C71—C76—H76	119.9
C41—C46—H46	120.2	C75—C76—C71	120.1 (5)
C41—C46—C45	119.7 (3)	C75—C76—H76	119.9
C45—C46—H46	120.2	C12—C13—H13	120.0
C19—C20—H20	119.9	C14—C13—C12	120.0 (5)
C19—C20—C21	120.2 (4)	C14—C13—H13	120.0
C21—C20—H20	119.9	C74—C75—H75	119.8
C11—C16—H16	119.8	C76—C75—C74	120.5 (5)
C15—C16—C11	120.4 (4)	C76—C75—H75	119.8

Symmetry codes: (i) −*y*+1, *x*−*y*+1, *z*; (ii) −*x*+*y*, −*x*+1, *z*.
